# Optimization of a metatranscriptomic approach to study the lignocellulolytic potential of the higher termite gut microbiome

**DOI:** 10.1186/s12864-017-4076-9

**Published:** 2017-09-01

**Authors:** Martyna Marynowska, Xavier Goux, David Sillam-Dussès, Corinne Rouland-Lefèvre, Yves Roisin, Philippe Delfosse, Magdalena Calusinska

**Affiliations:** 1 Luxembourg Institute of Science and Technology, 41 rue du Brill, L-4422 Belvaux, Luxembourg; 2 Institute of Research for Development - Sorbonne Universités, Institute of Ecology and Environmental Sciences - Paris, U242, 32 avenue Henri Varagnat, F-93140 Bondy, France; 3 University Paris 13 - Sorbonne Paris Cité, Laboratory of Experimental and Comparative Ethology, EA4443, 99 avenue Jean-Baptiste Clément, F-93430 Villetaneuse, France; 4 Université Libre de Bruxelles, 50 Avenue F.D. Roosevelt, B-1050 Brussels, Belgium

**Keywords:** Carbohydrate-active enzymes (CAZymes), Isoptera, Metatranscriptomics, Microbiome, mRNA enrichment, Termite gut

## Abstract

**Background:**

Thanks to specific adaptations developed over millions of years, the efficiency of lignin, cellulose and hemicellulose decomposition of higher termite symbiotic system exceeds that of many other lignocellulose utilizing environments. Especially, the examination of its symbiotic microbes should reveal interesting carbohydrate-active enzymes, which are of primary interest for the industry. Previous metatranscriptomic reports (high-throughput mRNA sequencing) highlight the high representation and overexpression of cellulose and hemicelluloses degrading genes in the termite hindgut digestomes, indicating the potential of this technology in search for new enzymes. Nevertheless, several factors associated with the material sampling and library preparation steps make the metatranscriptomic studies of termite gut prokaryotic symbionts challenging.

**Methods:**

In this study, we first examined the influence of the sampling strategy, including the whole termite gut and luminal fluid, on the diversity and the metatranscriptomic profiles of the higher termite gut symbiotic bacteria. Secondly, we evaluated different commercially available kits combined in two library preparative pipelines for the best bacterial mRNA enrichment strategy.

**Results:**

We showed that the sampling strategy did not significantly impact the generated results, both in terms of the representation of the microbes and their transcriptomic profiles. Nevertheless collecting luminal fluid reduces the co-amplification of unwanted RNA species of host origin. Furthermore, for the four studied higher termite species, the library preparative pipeline employing Ribo-Zero Gold rRNA Removal Kit “Epidemiology” in combination with Poly(A) Purist MAG kit resulted in a more efficient rRNA and poly-A-mRNAdepletion (up to 98.44% rRNA removed) than the pipeline utilizing MICROBExpress and MICROBEnrich kits. High correlation of both Ribo-Zero and MICROBExpresse depleted gene expression profiles with total non-depleted RNA-seq data has been shown for all studied samples, indicating no systematic skewing of the studied pipelines.

**Conclusions:**

We have extensively evaluated the impact of the sampling strategy and library preparation steps on the metatranscriptomic profiles of the higher termite gut symbiotic bacteria. The presented methodological approach has great potential to enhance metatranscriptomic studies of the higher termite intestinal flora and to unravel novel carbohydrate-active enzymes.

**Electronic supplementary material:**

The online version of this article (10.1186/s12864-017-4076-9) contains supplementary material, which is available to authorized users.

## Background

Termites are eusocial insects that are of special scientific and industrial interest due to their ability to decompose lignocellulosic biomass, which is the most abundant biopolymer on Earth [1]. They are successful in diverse ecosystems from wet tropical forests to dry savannahs. Termites can feed on vegetal material of various levels of humification, including soil, wood, litter, lichen, etc. [2]. Unlike lower termites that live in symbiosis with eukaryotic flagellates, higher termites mostly rely on their gut prokaryotes [3] and, in the case of Macrotermitinae, on an exo-symbiosis with fungus [4], to help them digest lignocellulose. In addition, higher termites themselves can secrete cellulases and other carbohydrate-active enzymes (CAZymes) by their midgut epithelium [5]. Thanks to these adaptations, their efficiency of lignin, cellulose and hemicellulose decomposition exceeds that of other lignocellulose utilizing systems, e.g. ruminants or fungi alone [6]. However, from an industrial perspective, the termite gut is a too complex environment to be mimicked, due to its structured microenvironments that differ in physicochemical conditions and the microbial processes that they accommodate [3]. Nevertheless, the examination of termite symbiotic microbes should reveal interesting enzymes or enzymatic cascades capable of hydrolysing a broad range of chemical bonds, which is of primary interest for the industry. While main part of the research has focused on endogenous endoglucanases of termites and on cellulases originating from termite gut flagellates, enzymes of bacterial origin have received much less attention [3]. Moreover, the complete loss of flagellates in higher termite means that they have to rely on different strategies to thrive and to digest lignocellulose. Indeed, except for the members of the subfamily Macrotermitinae [4, 7] all other subfamilies of higher termites are independent of fungal symbionts, therefore in addition to their own enzymes they rely on cellulolytic bacterial partners for their survival and for an efficient lignocellulose digestion. Interestingly, significant reduction in cellulase activity has been observed in hindguts of *N. takasagoensis* after antibiotic treatment [8]. Moreover, significant enzymatic activities targeting cellulose and hemicellulose, detected in the symbiotic metagenome of the higher termite *Nasutitermes corniger* hindgut, further support the crucial role of bacteria in lignocellulose digestion and termite feeding [9].

Studying naturally evolved biomass-degrading microbial communities, with the use of the novel high-throughput DNA sequencing technologies (metagenomics), presents a new strategy to identify novel enzymes with potentially high activities. However, besides the considerable sequencing depth of the different studies (e.g. [10]), only a subset of genes present in the metagenome could be assembled, as further indicated by a rarefaction analysis. The average frequency of target genes in microbial genomes is lower than two glycoside hydrolases per bacterial genome [11]. By contrast, limited metatranscriptomic reports (high-throughput mRNA sequencing) highlighted the high representation and overexpression of cellulose and hemicellulose degrading genes in the termite hindgut digestome, indicating the potential of metatranscriptomics to discover new CAZymes in this specific environment (e.g. [9]). The importance of the de novo transcriptome assembly to reconstruct and functionally characterize abundant transcripts originating from the subsidiary species not well represented in the corresponding metagenomic data sets has been already discussed for termite microbiome (highly expressed glucose hydrolases were identified solely from the de novo assembled metatranscriptome and not from the accompanying metagenome [9]), as well as for other environments, including microbial communities in the deep sea [12] and plankton communities inhabiting surface and subpycnocline waters [13].

In the case of the higher termite system, mainly amplicon-based and metagenomic studies have been applied up to date [14–16]. So far, mRNA sequencing has mainly been employed to study the expression profiles of host or lower termite eukaryotic symbionts [17, 18]. The metatranscriptomics of the higher termite gut prokaryotic symbionts are in their infancy with only one published study [9]. One of the reasons might be that eukaryotic poly-A mRNA is relatively easier to enrich with oligo-T affinity methods [19, 20]. Other hindering factors are the low average ratio of the prokaryotic mRNA in comparison to the other bacterial and host RNA species (e.g. rRNA, tRNA), short life-time of mRNA and the restrictions of the commercially available kits to efficiently enrich for bacterial mRNA [21]. The latter limitation is particularly important in the case of non-model organisms such as termites, which have only two representatives with sequenced genomes [22, 23]. The employed sampling strategy of the termite gut (e.g. the choice between sampling and sequencing the whole termite gut [WG], luminal fluid [LF] or separate gut compartments) is another factor that should be addressed in order to minimise the co-extraction of unwanted macromolecules (e.g. contaminant host DNA and RNA). However, while most of the bacterial gut symbionts live freely suspended in the luminal fluid, some might be attached to the hindgut wall or food particles [3, 24]. Therefore, the prokaryote-oriented metatranscriptomic study should be carefully designed to balance between the best sampling strategy providing the true representation of the symbionts diversity, and the optimization of the co-extraction of unwanted macromolecules.

In this study, we aimed at optimizing the framework for an accurate termite gut prokaryote-oriented metatranscriptomics that can be further extended to other multi-omics approaches, e.g. amplicon-sequencing and metagenomics. We have focused on two aspects. Firstly, by applying 16S rRNA gene and mRNA sequencing, we have compared the influence of two sampling strategies, whole termite gut versus the luminal fluid content, on the diversity and transcriptomic profiles of the gut-associated microbes. Secondly, we have evaluated the best combination of commercially available kits for an optimised bacterial mRNA enrichment and high-throughput sequencing, in search for novel CAZymes of potential scientific and industrial interest. As a result, we propose a fully-optimized framework for an efficient metatranscriptomic analysis of the termite gut symbiotic communities.

## Methods

### Samples collection

**Fig. 1:**
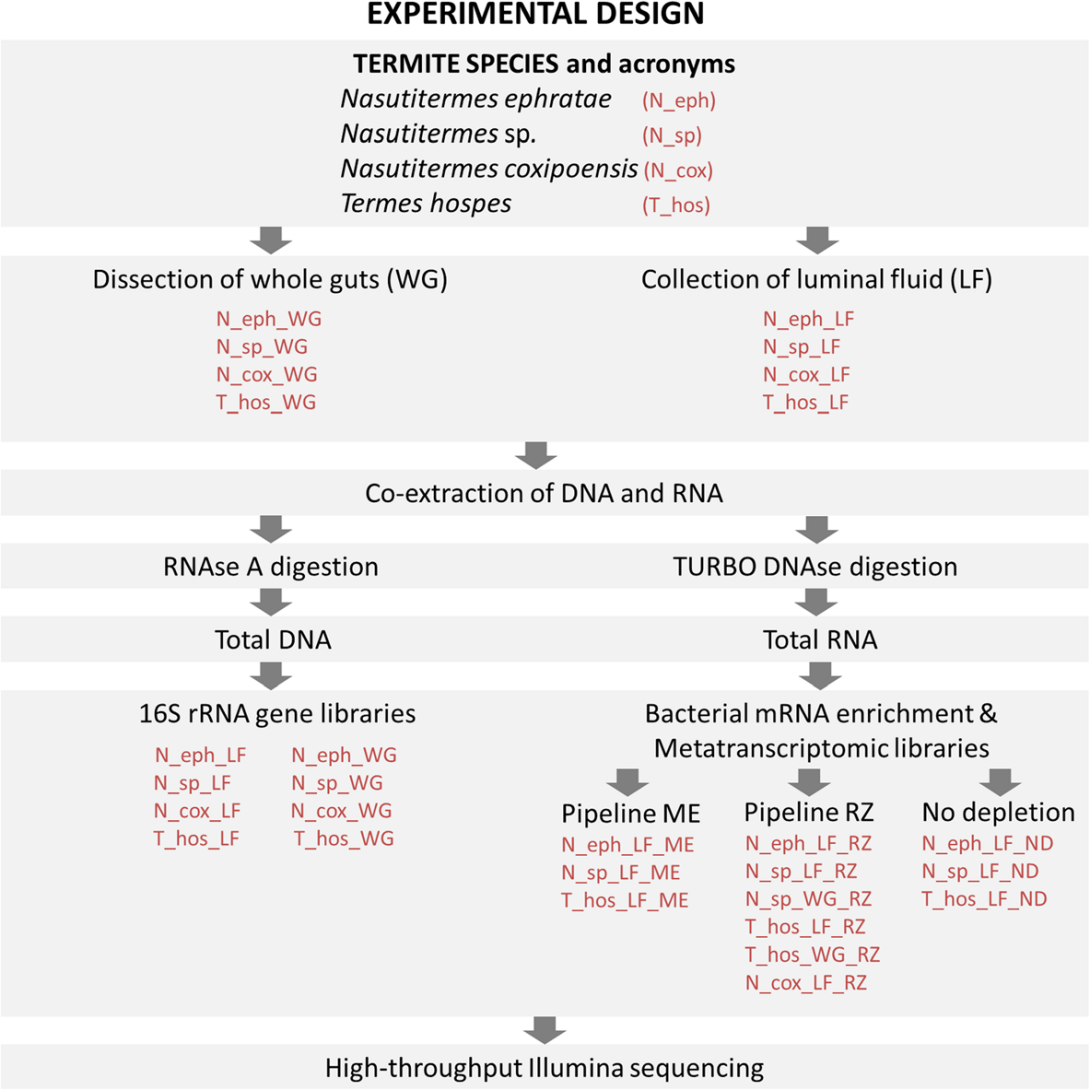
Overview of the experimental design and samples included in the study. LF – luminal fluid extract, WG – whole guts; Pipeline ME included the use of MICROBEnrich Kit (Ambion) followed by MICROBExpress Kit (Ambion), Pipeline RZ consisted of Ribo-Zero Gold rRNA Removal Kit “Epidemiology” (Illumina) in combination with Poly(A) Purist MAG Kit (Ambion)

The schematic experimental design is shown in Fig. 1. Wild mature workers were collected from the nest of grass-feeding *Nasutitermes coxipoensis* (*N. coxipoensis*), located in a tropical savannah in French Guiana and from three wood-fed termite colonies *Nasutitermes ephratae* (*N. ephratae*), *Nasutitermes* sp*.* (*N.* sp.) *and Termes hospes* (*T. hospes*) maintained in the laboratory at the IRD in Bondy-France. Details concerning the origin of the nests are specified in Additional file 1: Table S1. All collected worker individuals were cold-immobilized, surface-sterilized with 80% ethanol and decapitated. For all samples, the dissection was performed in two manners. Either whole guts or the luminal fluid was collected (Fig. 1). In the latter case, a sterile tip was used to pinch the gut in order to release its luminal content. The gut was further flushed thoroughly with 20 μL of Phosphate-Buffered Saline solution (PBS 1X, pH 7.4, Ambion) or RNAlater® Stabilization Solution (Ambion) and the luminal fluid was collected. Approximately 30 guts or luminal contents were pooled together to form composite samples of whole guts or luminal fluid. Samples were stored at −80 °C until further processing.

### Termite species identification

Termite species were primarily identified by morphological features. Additionally, DNA was extracted from the heads of the collected workers using AllPrep DNA/RNA Micro Kit (Qiagen) following the manufacturer’s instructions with minor modifications. The bead-beating with sterile milling beads (2 × 5 mm and 5 × 2 mm) at 15 Hz for 2 min was used to lyse the cells. Subsequently, termite identification was performed by analysing the sequence of the CO-II (cytochrome oxidase subunit 2) marker gene, as previously described [25]. The nucleotide sequences were submitted to GenBank database and are available under accession numbers MF176392 to MF176395.

### Nucleic acids extraction from gut microbiome of termites

Samples were centrifuged at 5000 g for 5 min to pellet the cells and the excess of the storage solution was removed prior the isolation. Cell pellets were resuspended in 200 μL of 1× PBS. DNA and RNA for all the samples were simultaneously co-extracted using the PowerViral Environmental RNA/DNA Isolation Kit (MO-BIO) following manufacturer’s instructions. This commercially available kit enables isolation of viral and bacterial DNA and RNA from wastewater, stool, biosolids and gut material. The chemical cell lysis was supplemented with mechanical bead-beating for 2 min at 20 Hz using 0.1 mm glass beads, to assure the disruption of most bacterial cells. Eluents were divided into two aliquots. The first half was treated with 1 μL of 10 μg/mL RNase A (Sigma) at room temperature (RT) for 30 min. The second half was treated with TURBO DNA-free Kit (Invitrogen), following manufacturer’s instructions, except for the step for which the use of DNAse Inactivation reagent was replaced by purification of RNA using Agencourt RNAClean XP Kit (Beckman Coulter). DNA and RNA were quality-assessed using respectively agarose gel electrophoresis and Bioanalyser RNA 6000 Pico Kit (Agilent; Additional file 2: Figs. S1 to S4). In general, LF RNA preparations were of better quality than WG, probably due to the contamination with the partially degraded termite genetic material in the second case. The DNA and RNA concentrations were quantified with Qubit dsDNA HS Assay Kit and Qubit RNA HS Assay Kit (Invitrogen), respectively. Eluted RNA was further divided into aliquots to represent a uniform starting material for downstream treatments and controls. DNA was stored at −20 °C and RNA at −80 °C until further processing.

### Bacterial 16S rRNA gene amplicon sequencing

The bacterial 16S rRNA gene libraries were prepared using modified primers S-D-Bact-0909-a-S-18 and S-*-Univ-*-1392-a-A-15 [26], according to the Illumina platform-compatible approach described elsewhere [27]. Briefly, the first round polymerase chain reaction (PCR) was carried out using Q5® Hot Start High-Fidelity 2X Master Mix (New England Biolabs) in triplicate reactions, consisting of 1 ng of template DNA, 0.4 μM primers and 1 mg/mL BSA (Sigma), with cycling conditions as follows 98 °C 30 s, 22 cycles of: 98 °C 5 s, 58 °C 30 s, 72 °C 30 s and final extension at 72 °C for 2 min. After pooling the triplicate PCR products, these were further beads-purified (Agencourt AMPure XP, Beckman Coulter) and the concentration was assessed with Qubit dsDNA HS Assay Kit. One ng of each purified product was used in a second round PCR, together with 5 μL of each of the index primers (Nextera XT Index Kit V2 Set C, Illumina) per sample. Reaction conditions were as described above, except for the annealing temperature that was set to 55 °C and the number of cycles to 8. Purified libraries were pooled together in equimolar ratios and the pool was quantified by quantitative polymerase chain reaction (qPCR) using KAPA SYBR FAST Universal qPCR Kit (KapaBiosystems). The pool was mixed with 2% of PhiX control (Illumina) and sequenced using MiSeq Reagent Kit V3–600 on the Illumina Platform. The obtained sequence reads were de-multiplexed, quality trimmed and assigned to operational taxonomic units (OTUs) at 97% similarity with Usearch (v7.0.1090_win64) pipeline [28]. Taxonomic affiliation of normalized reads was performed with the SILVA database v.123 [29] using mothur software v.1.38.0 [30]. Statistical analyses were performed using mothur and R environment [31] on the sequences annotated of prokaryotic origin.

### Bacterial mRNA enrichment

Two pipelines for the bacterial mRNA enrichment were compared and applied to selected RNA samples (Fig. 1). The first pipeline (further referred to as “Pipeline ME”) included the use of MICROBEnrich Kit (Ambion) followed by MICROBExpress Kit (Ambion). Kits were used following manufacturer’s instructions, except for the final bacterial mRNA precipitation, which was replaced by the purification step with Agencourt RNAClean XP Kit (1.8× volume of beads per 1× volume of sample). The second pipeline (further referred to as “Pipeline RZ”) consisted of Ribo-Zero Gold rRNA Removal Kit “Epidemiology” (Illumina) in combination with Poly(A)Purist MAG Kit (Ambion). The original procedure of the second kit was modified to separately recover bacterial non-poly-A-mRNA. Briefly, following the last step of the Ribo-Zero Gold kit protocol, where mRNA was purified with Agencourt RNAClean XP Kit and eluted in water, 60 μL of the elution reaction was added to the 2× Binding buffer from the Poly(A) Purist MAG Kit and manufacturer’s protocol was followed until the point where Oligo(dT) MagBeads were captured on magnetic stand. Here, the supernatant containing the desired bacterial mRNA and depleted of poly-A-eukaryotic mRNA was retained. The collected supernatant was further purified with Agencourt RNAClean XP Kit and prokaryotic mRNA was eluted in water. Aliquots of the non-depleted (ND) RNA were also maintained as control. In continuation, based on the obtained results, Pipeline RZ was retained to selectively enrich bacterial mRNA in the remaining samples to subsequently pursue the search for CAZymes transcripts.

### Metatranscriptomic analysis of termite gut bacteria

SMARTer Stranded RNA-Seq Kit (Clontech) was used according to the manufacturer’s instructions to prepare metatranscriptomic libraries. Bacterial mRNA, depleted of rRNA and poly-A mRNA, as well as non-depleted controls were used as inputs (Fig. 1). The quality of each library and size distribution were checked on Bioanalyzer using High Sensitivity DNA Kit (Agilent) and their concentrations were determined using KAPA SYBR FAST Universal qPCR Kit. Size distribution of prepared libraries ranged between 385 bp and 505 bp, with the average of 427 bp. Libraries were subsequently pooled together in equimolar concentrations (including 2% PhiX control) prior to sequencing with MiSeq Reagent Kit V3–600 on Illumina Platform (libraries were sequenced in three sequencing runs). Previous metatranscriptomic studies reported excellent correlation between the technical replicates concerning both the efficiency of rRNA removal and the transcriptomic profiles of the protein coding genes [32, 33]. Therefore, at the expense of technical replicates in our study we decided to evaluate a broader range of environmental samples. In total the sequencing effort resulted in over 112 M paired-end reads (nearly 17 GB of data). Average read length was 210 bp before and 182 bp after trimming. The obtained data was analysed according to the pipeline presented in Additional file 3: Figure S5. After initial quality trimming using CLC Genomics Workbench 9.0.1, reads were depleted of the remaining contaminating rRNA using SortMeRNA 2.0 [34]. Non-rRNA reads were used to perform three separate co-assemblies (Co-assembly_1, Co-assembly_2 and Co-assembly_3, for details see Additional file 4: Tables S2 to S5). For each library approximately 36.7% ± 6.1 of reads were mapped back to the obtained contiguous sequences (contigs) using “count paired reads as two” option implemented in CLC Genomics Workbench 9.0.1. Libraries in this study were considered free of DNA-amplification artefacts due to the lack of unspecific peaks on Bioanalyser elecropherogram of mRNA used as input for their preparation as well as due to the short average contigs lengths specific to the assembled mRNA reads (N50 = 313, N50 = 354 and N5 = 322 for co-assemblies 1, 2 and 3, respectively). Next, Bioconductor DESeq package implemented in R [35] was used to normalise the genes’ mapping counts to allow for the in-between samples comparisons and to obtain the relative abundance of the contigs. The co-assembled contigs were then used as input for Prodigal software [36] for genes prediction. Transcripts encoding for CAZymes were searched with the dbCAN [37] against a CAZy database [38]. For the taxonomic and functional annotation, assemblies were submitted to the IMG-MER [39], what additionally resulted in assignment of the contigs to clusters of orthologous groups of proteins (COG categories, [40]). Contigs carrying protein coding genes that were neither assigned of bacterial nor archaeal origin were excluded from further analysis. Statistical analyses were performed using R and R packages vegan [41] and MASS [42].

## Results and discussion

### Analyses of the higher termite whole guts and luminal fluid contents provide comparable profiles of bacterial community structures and transcription levels of protein coding genes

For the purpose of the omic studies different sampling strategies have been used to collect termite gut material, making the comparison of the insect gut symbiotic communities between the different studies challenging. Depending on the study design, either whole termite guts or specific gut compartments have been targeted. For instance, in their metagenomic and functional study Warnecke et al. investigated the luminal content of a specific compartment, collected after incising the hindgut with a needle [14]. In another study, whole termite guts were collected for the amplicon-based characterisation of gut symbionts [43]. The use of a whole gut seems proper when the genetic material of interest is further specifically amplified. Yet, in the case of the contamination-sensitive metagenomics and metatranscriptomics, it is crucial to have as little contaminant DNA and rRNA as possible. Co-sequencing of the non-desired genetic fragments reduces the sequencing depth, e.g. leading to the underdetection of the less abundant transcripts. In the past, this issue has been addressed by applying a mild-trypsin digestion to the collected whole guts to release microbial cells and to remove the excess of the host material [44]. In the case where bacteria cohabit the termite gut with protists (lower termites), an additional challenge is to separate bacterial cells not only from the termite host but also from the micro-eukaryotes. To that purpose, in another metagenomics study Do et al. employed different centrifugation steps in order to exclusively select microbial cells [45]. Nevertheless, it is questionable to what extent such procedures alter the metatranscriptomic profile, especially as bacterial mRNA is unstable and should be quickly and properly preserved [21]. In addition, bacterial cells may have different spatial distributions. They are not only free living in the luminal fluid but may also be attached to the termite gut walls, to fibre-material, or located inside the intracellular compartments of the endosymbiotic protists in the case of lower termites [24, 46].

#### 16S rRNA amplicon sequencing

**Fig. 2:**
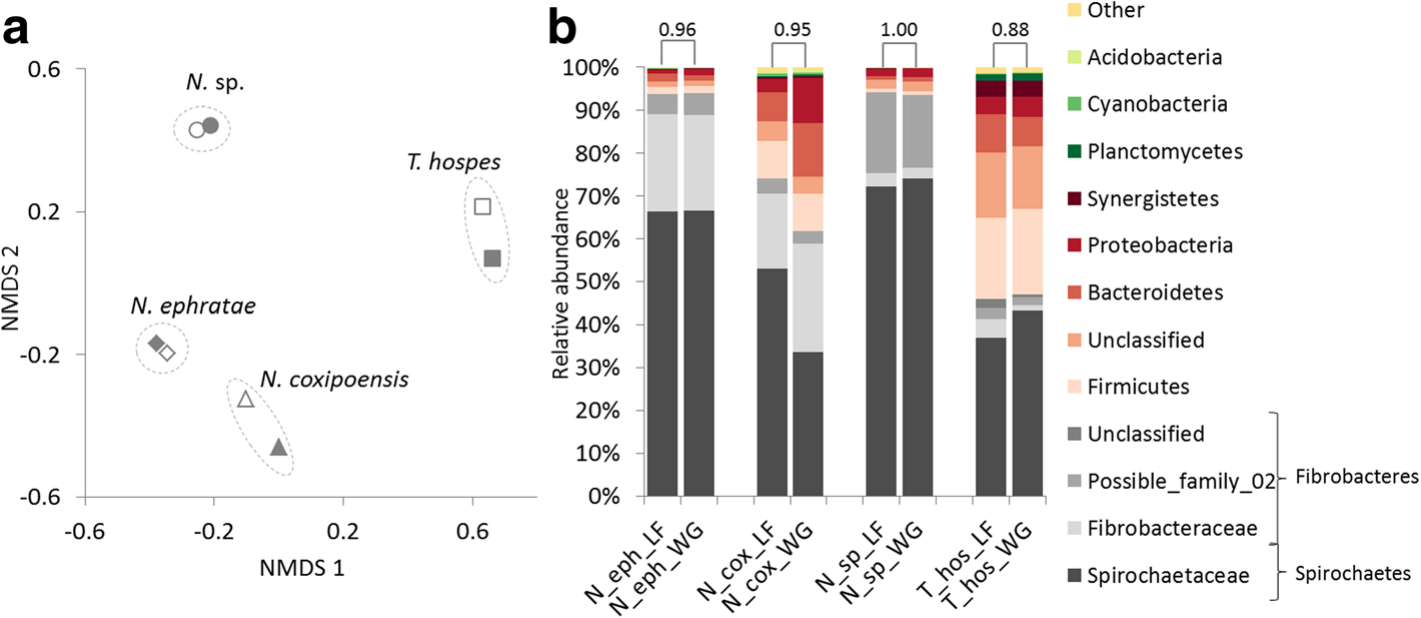
Results are based on the 16S rRNA gene amplicon high-throughput sequencing. **a** NMDS ordination of the calculated Bray-Curtis dissimilarities in bacterial community structures at the OTU level (stress value 0.15, R^2^ = 0.89). Empty and full symbols represent the luminal fluid (LF) and whole guts (WG) sampling strategies, respectively, for the different termite species. **b** Taxonomic distribution of bacterial OTUs into the different phyla (assigned according to the SILVA database v.123). Relative abundances were derived based on number of normalized reads assigned to specific OTUs. Numbers on the top of each bar pair represent the calculated Spearman correlation coefficient between pairs of samples. All levels of significance are <0.001

To reliably interpret the metatranscriptomic results, we first investigated the impact of the termite gut sampling strategies, here the use of the whole termite guts versus the luminal fluid extracts. To that purpose, we first compared bacterial communities in eight termite gut samples collected either as whole termite guts or luminal fluids from four different termite species (Fig. 1). The high-throughput 16S rRNA gene amplicon sequencing resulted in a total of 918,176 normalised reads that were assigned to 1646 bacterial OTUs. The calculated rarefaction curves were flatter to the right for most of the samples, indicating that further sampling would only yield a few additional species (Additional file 5: Figure S6). The calculated richness and diversity indices were consistent between the LF and WG preparations and the bacterial richness was twice higher for *T. hospes* in comparison to the *Nasutitermes* species (Additional file 5: Table S6). Also, the non-metric multidimensional scaling (NMDS) ordination of the calculated Bray-Curtis dissimilarities in bacterial community structures, pointed towards nearly identical bacterial community profiles for both LF and WG collected from the same termite species (Fig. 2a). Similar bacterial distribution into the different bacterial phyla for both LF and WG was further supported by the high values of the calculated Spearman correlations (correlation coefficients ranging from 0.88 to 1.00; Fig. 2b). These results indicate a minor impact of the termite gut sampling strategy on the microbial representation. The microbiomes of the three termite species from the genus *Nasutitermes* were over-represented by Spirochaetaceae family (> 50% of total 16S rRNA reads), followed by representatives of Fibrobacteres phylum. In the case of *T.hospes*, the most dominant phyla were Spirochaetes, Firmicutes and Bacteroidetes. These results remain consistent with the previously published reports for the wood- and soil-feeding termites [47].

#### Metatranscriptomics

**Fig. 3:**
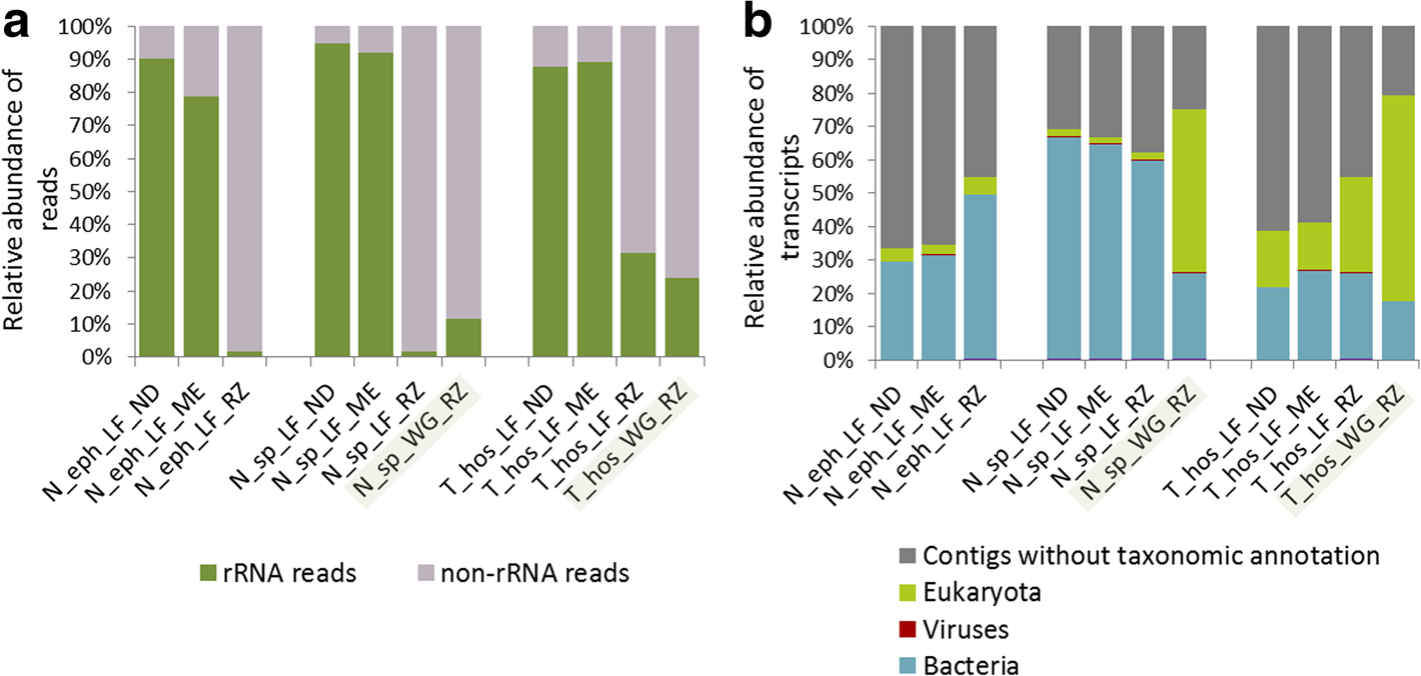
Comparison of the efficacy of the two studied metatranscriptomic pipelines versus the non-depleted control. The performance of two pipelines, RZ and ME, was tested for three termite species: *N. ephratae*, *N.* sp. and *T. hospes.* LF corresponds to luminal fluid and WG to whole termite gut RNA preparations. **a** Proportion of rRNA reads detected in the different metatranscriptomic libraries for the two studied pipelines and the non-depleted control. WG sampling strategy was only assessed with the pipeline RZ for *T. hospes* and *N.* sp. preparations. **b** Relative abundance of transcripts with no taxonomic assignment and assigned as of prokaryotic (archaea and bacteria), eukaryotic or viral origin

**Fig. 4:**
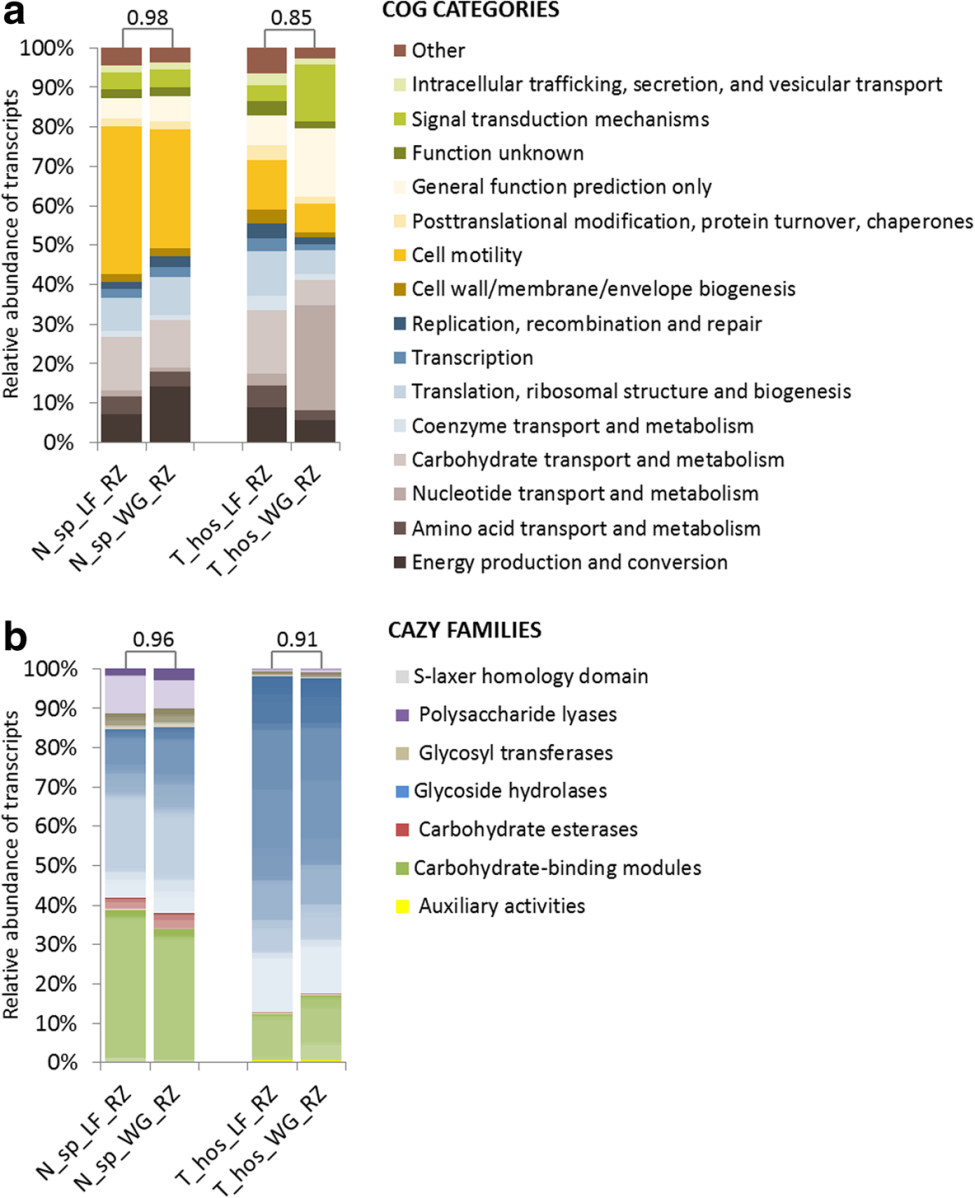
Comparison of the prokaryotic metatranscriptomic profiles for luminal fluid (LF) and whole termite guts (WG) RNA preparations. **a** Prokaryotic gene transcripts annotated to Clusters of Orthologous Group (COG) categories. **b** Distribution of prokaryotic transcripts to CAZy families (only contigs with e-value <1e^−18^ and coverage >0.35 are shown). Different families within the same class are distinguished by the colour gradient. Numbers on the top of each bar pair represent the calculated Spearman correlation coefficient between pairs of samples. All levels of significance are <0.001

To evaluate the impact of the sampling strategy on prokaryotic transcriptomic profiles, we further applied the optimised metatranscriptomic protocol (the protocol RZ is described in a paragraph below) to WG and LF RNA extracts from two representative termite species, *N.* sp*.* and *T. hospes*. As a result, the amount of the detected rRNAs in WG versus LF RNA preparations was higher in the case of *N*. sp., while the situation was inversed for *T. hospes*. In both cases detected rRNAs remained at relatively low levels (Fig. 3a; Additional file 4: Table S3). Following the taxonomic affiliation, for the WG samples up to 61.9% and 48.8% of the reconstructed mRNA transcripts were of eukaryotic origin, for *T. hospes* and *N*. sp. libraries, respectively (Fig. 3b). In the case of LF, poly-A-mRNA transcripts were much less abundant for *N*. sp. (2.2% of reconstructed mRNA transcripts) but the depletion was less successful for LF of *T. hospes* (28.3%). Concerning *T. hospes,* a reason behind the altered metatranscriptomic profiles might be the slightly lower quality of the RNA preparations*.* However, previous study using both an artificially fragmented RNA from a mock microbial community and from a stool sample demonstrated a very minor influence of a partially degraded RNA on rRNA depletion and resulting mRNA profiles [32]. On the other hand, another study provided evidence for the lower oligo-dT affinity and hybridization-based removal efficiency of unwanted RNA species in the case of degraded RNA preparations [21]. Based on the prokaryotic gene transcripts annotations to COG categories [40], the generated transcriptomic profiles for the WG and the LF preparations for the same sample were more similar for *N*. sp. than *T. hospes* (Spearman correlation coefficients respectively 0.98 and 0.85; Fig. 4a). Due to the fact that the calculated gut bacterial diversity was twice as high for *T. hospes* in comparison to *N*. sp. (Additional file 5: Table S6), insufficient sequencing might have impacted the overall COGs distribution for *T. hospes* (Additional file 4: Table S2); especially if multiple eukaryotic transcripts were present. Another reason behind the observed minor differences in the corresponding metatranscriptomic profiles between the WG and LF samplings may arise from the differential expression of genes in the case of the planktonic and gut wall attached (biofilm-forming) bacteria. Depending on the gut shape and the amount of paunch filaments, this difference might be more or less pronounced. The difference in the gene expression profiles and physiological distinction for the same bacterium showing planktonic and biofilm-forming lifestyles has previously been documented for example in the microarray-based study for *Clostridium acetobutylicum* [48] and transcriptomic study for *Desulfovibrio vulgaris* sp*.* [49]. Interestingly, deeper analysis indicated highly similar CAZymes profiles for the two sampling strategies, with the calculated Spearman correlation coefficients between 0.96 and 0.91 respectively for *N*. sp. and *T. hospes* (Fig. 4b). In the context of the termite gut, CAZymes are one of the most expressed genes, therefore lower sequencing depth should not skew significantly their expression profiles (see the section concerning the CAZymes analysis).

Above results demonstrate high overall efficacy of the prokaryotic mRNA enrichment when using the LF sampling strategy. In the case of WG RNA preparation, the efficacy of the removal of the highly abundant host poly-A mRNAs is much lower and may compromise the expected sequencing outcome. The rRNA depletion rate was higher for LF than WG for *N*. sp. but lower for *T. hospes*, indicating that it might be species-related or sensitive to the initial RNA quality. While these preliminary results do not provide any strong evidence of any systematic skewing caused by using the WG over the LF preparations (except for a reduced sequencing depth due to the presence of host poly-A mRNAs), future metatranscriptomic studies of termite gut prokaryotic communities using a higher number of different samples would help to verify this outcome. For the purpose of this study, we used the LF RNA preparations to evaluate proposed pipelines of bacterial mRNA enrichment.

### Efficient enrichment of bacterial mRNA in total RNA extracts from termite guts is achievable using a combination of commercially available kits

Unlike for model organisms such as human, mouse or rat, there are no commercially available kits specifically optimized to target the prokaryotic mRNA of the higher termite gut system. Firstly, prokaryotic RNA should be efficiently separated from the host RNA species (both rRNA/tRNA and poly-A mRNA should be depleted). Secondly, prokaryotic rRNA/tRNAs should be removed for an efficient enrichment of the prokaryotic mRNAs in the sample. Up to now, solely the use of the MICROBExpress Bacterial mRNA enrichment Kit has been evaluated to study the metatranscriptome of a higher termite prokaryotic gut symbionts [9]. There has been no other study in the context of the higher termite gut microbiome that could serve as a guideline to the researchers to efficiently perform metatranscriptomic studies. This lack of methodology might be the primary reason for the scarce metatranscriptomic reports concerning the higher termite gut symbionts in comparison to their lower termite counterparts [18, 50–52].

Therefore, in this study, we have evaluated two pipelines for an efficient bacterial mRNA enrichment from termite gut RNA extracts in order to provide the best “wet-lab” methodology to encourage metatranscriptomic studies of the higher termite bacterial symbionts. The first pipeline (“pipeline ME”) makes use of the MICROBEnrich Kit combined with the MICROBExpress Kit. While the first kit has been optimized by the manufacturer for the removal of mammalian 18S and 28S rRNA and poly-mRNA (previously tested on human, rat and mouse), the second one removes bacterial 16S and 23S rRNAs. The second pipeline (“pipeline RZ”) uses the Ribo-Zero Gold rRNA Removal Kit “Epidemiology” in combination with the Poly(A)Purist MAG Kit. The first product is designed to remove eukaryotic cytoplasmic and mitochondrial rRNA as well as bacterial rRNA. The second kit further purifies the poly-A mRNAs, but here the protocol was adapted to target specifically bacterial mRNAs. Still, if the co-purification of the poly-A mRNA is of interest, it can be simultaneously recovered by applying the original Poly(A) Purist MAG Kit protocol.

Both pipelines were applied to the three sets of LF RNA extracts from *N. ephratae*, *N.* sp*.* and *T. hospes* (Fig. 1). Following the sequencing and data processing, the percentage of rRNA reads detected for the libraries prepared according to the pipeline RZ was very low in comparison to pipeline ME and ND controls (total RNA sequencing; Fig. 3a). It accounted for 1.56%, 1.64% and 31.43% respectively for N_eph_LF_RZ, N_sp_LF_RZ and T_hos_LF_RZ libraries. The very high accuracy of the mRNA enrichment in the case of the pipeline RZ was further confirmed by the annotation of above 99.9% of the assembled gene transcripts to protein coding genes (Additional file 4: Table S4).

In the context of the termite gut system, the performance of the pipeline RZ definitely exceeds that of the otherwise used MICROBExpress kit. Indeed, the application of the latter kit in the previously published metatranscriptomic report only slightly enriched bacterial mRNA, resulting in a dramatic decrease of the sequencing depth, as above 86% and 87% of the sequencing reads were of rRNA origin for the two investigated termite species, *Amitermes wheeleri* and *Nasutitermes corniger* [9]. Moreover, the MICROBExpress kit is designed to only remove prokaryotic rRNAs, and it does not eliminate the eukaryotic rRNAs which might still be present in the prepared library. By contrast, the high efficiency of the Ribo-Zero Gold rRNA Removal Kit used in the pipeline RZ has been previously documented for pure bacterial cultures [33] and more complex microbial communities [32]. In addition, in the case of the mosquito metatranscriptome the application of the RiboZero Gold rRNA Removal Kit resulted in the depletion of the 28S and 18S rRNAs to the level of 2.5% and 29.5% of total sequencing reads in the resulting libraries [53]. The application of the same library preparation strategy to study the metatranscriptome of stool samples allowed for enriching the prokaryotic mRNAs to the level of 30 to 98% of total sequencing reads, depending on the initial sample integrity state [21]. In the study of *Wolbachia-Drosophila* lateral gene transfer [54] the use of RiboZero removal kit- Human/mouse/rat (which belongs to the same family of kits, but does not target neither mitochondrial nor bacterial rRNA) allowed for the 98% reduction of insect rRNA and 6.2-fold increase in the detection of mRNA transcripts.

In our study, further taxonomic affiliation of the rRNA-free mRNA transcripts revealed a high proportion of non-assigned transcripts, on average 49.5% ± 14.0 for the studied LF samples. For the two *Nasutitermes* species studied prokaryotic transcripts (mostly bacterial and to some extent archaeal) were more abundant than eukaryotic and virus-assigned genes (Fig. 3b). For both ME and RZ pipelines, the level of the poly-A transcripts in LF samples remained in the range of 1.8 to 5.0% respectively for *N. ephratae* and *N.* sp. Higher percentage of poly-A mRNAs was detected by the two pipelines and in the ND control for LF of *T. hospes* (depending on the pipeline between 14.1 and 28.3% of all reconstructed mRNA transcripts, and up to 16.6% in case of ND control). While sample-inherent factors (termite species-related factors, e.g. lower hybridization efficacy due to probe sequence mismatches, higher overall amount of eukaryotic transcripts, etc.) cannot be excluded, the oligo-dT affinity and hybridization-based removal of unwanted RNA species have also been shown to depend on the sample quality [21].

### Both RZ and ME mRNA enrichment pipelines retain the original prokaryotic metatranscriptomic profiles

**Fig. 5:**
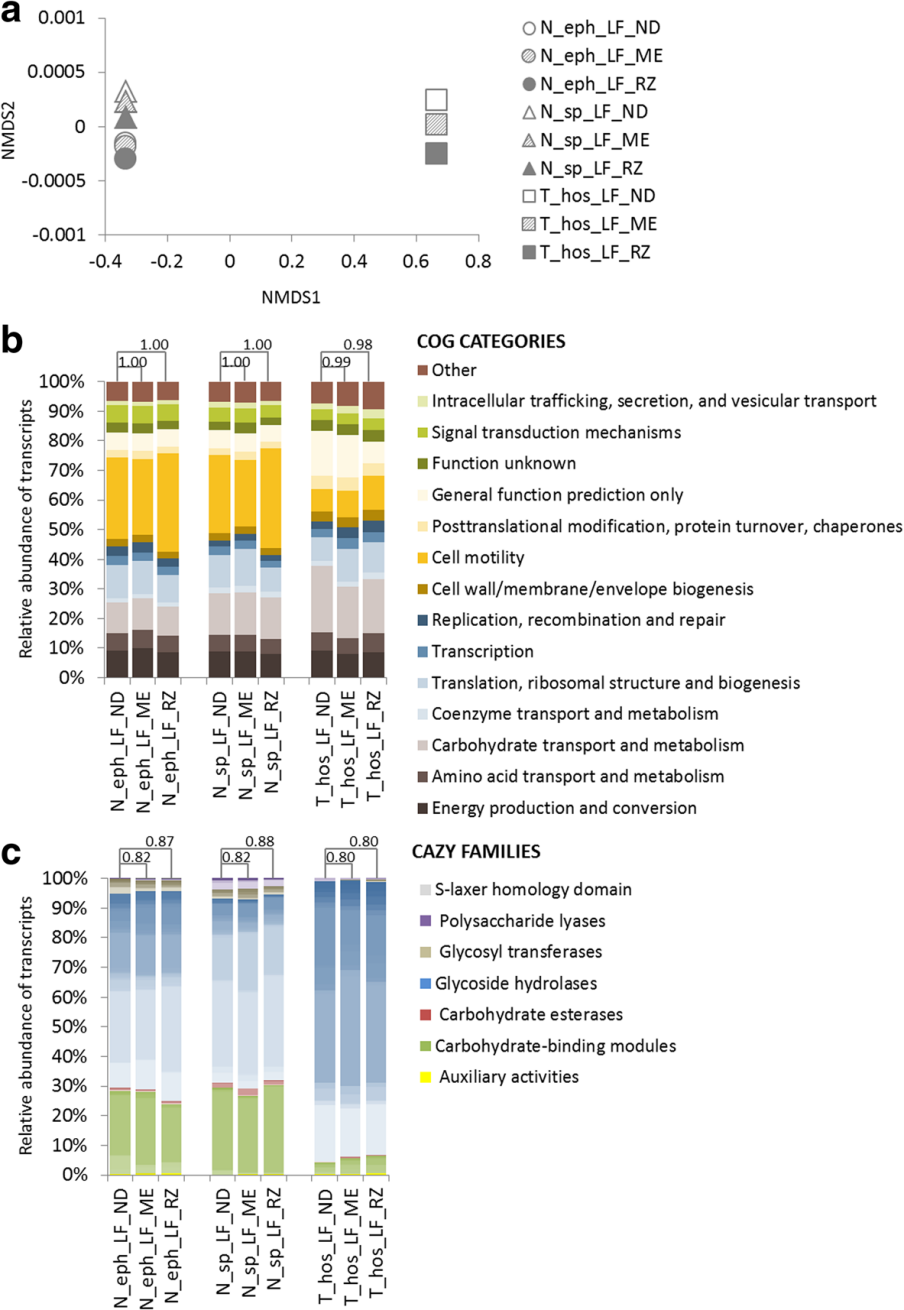
Comparison of the metatranscriptomic profiles for two prokaryotic mRNA enrichment pipelines versus the non-depleted controls. ND - libraries not preceded by mRNA enrichment, ME - libraries prepared with Pipeline ME, RZ - libraries prepared with Pipeline RZ. **a** NMDS ordination of the calculated Bray-Curtis dissimilarities of the resulting metatranscriptomic profiles (stress value <0.20). **b** Distribution of the prokaryotic gene transcripts annotated to the different COG categories. **c** Distribution of the prokaryotic contigs annotated to the different CAZy families (only contigs with e-value <1e^−18^ and coverage >0.35 have been used)**.** Numbers on the top of each bar pair represent the calculated Spearman correlation coefficient between the two studied mRNA enrichment pipelines and ND control. All levels of significance are <0.001

An ideal metatranscriptomic library preparation pipeline needs not only to efficiently enrich the bacterial mRNA but also to retain their original transcriptomic profiles. Therefore, in continuation we compared the resulting transcriptomic profiles between the RZ and ME pipelines and towards the ND RNA control (total RNA sequencing) for two *Nasutitermes* (*N. ephratae* and *N.* sp*.*) and *T. hospes*. As a result, regardless of the applied mRNA enrichment pipeline, the profiles of gene transcripts for the libraries originating from the same termite species formed tight clusters on an NMDS graph (Fig. 5a). Additionally, the normalised distribution of assembled contigs and their annotation to COG categories resulted in virtually identical transcriptomic profiles (according to the very high Spearman correlation coefficient values in the range from 0.98 to 1.00; Fig. 5b), for the pipelines RZ and ME in relation to the non-depleted control sample. For *T. hopes*, even if the lower quality of the RNA preparation might have resulted in a decreased efficiency of the poly-A removal, it did not influence significantly the observed transcriptomic profiles of its gut prokaryotes. Additionally, both RZ and ME pipelines resulted in CAZymes profiles highly similar to the control sample for the three termite species studied (Fig. 5c). Similarly to our results, high correlation of Ribo-Zero depleted gene expression profiles with total non-depleted RNA-seq data has previously been shown for stool samples [32].

Taken together the above results, we conclude that the RZ pipeline has a superior performance to the ME pipeline in the context of the termite gut. Even though, both pipelines preserve well the metatranscriptomic profiles originally observed in non-depleted control RNA sample, enrichment of mRNA transcripts during the library preparation reduces the number of unwanted RNA reads being sequenced and therefore we strongly recommend the use of the RZ pipeline in the context of the higher termite gut symbionts metatranscriptomic studies.

### Diversity of CAZymes identified in the metatranscriptomes of the higher termite symbionts

By optimising the termite gut sampling strategy and metatranscriptomic library preparation pipelines, we aimed in this study at designing the best methodological framework for an improved metatranscriptomic analysis of the higher termite gut symbionts, with a special focus on their lignocellulolytic potential. Using the optimised approach (LF RNA preparation and the RZ pipeline), we finally compared the diversity of CAZymes in the metatranscriptomes of the gut symbiotic communities for the four termite species targeted in this study (Fig. 1). Importantly, for the four analysed metatranscriptomes, the COG category related to carbohydrate transport and metabolism was the second most highly represented (on average 14% ± 4 of all mRNA reads, Additional file 6: Figure S7), right after cell motility (26% ± 10). This result points towards the relatively high expression levels of different CAZymes in relation to other mRNA transcripts present in the metatranscriptome of the higher termite gut symbionts. Moreover, taking the advantage of the metatranscriptomic approach optimized in our study, we were able to identify multiple CAZymes even using the sequencing throughput provided by Illumina MiSeq platform; putatively lower than that of a typical metatranscriptomic study. This is a very important aspect in the case of some budget-limited research programs.

**Fig. 6:**
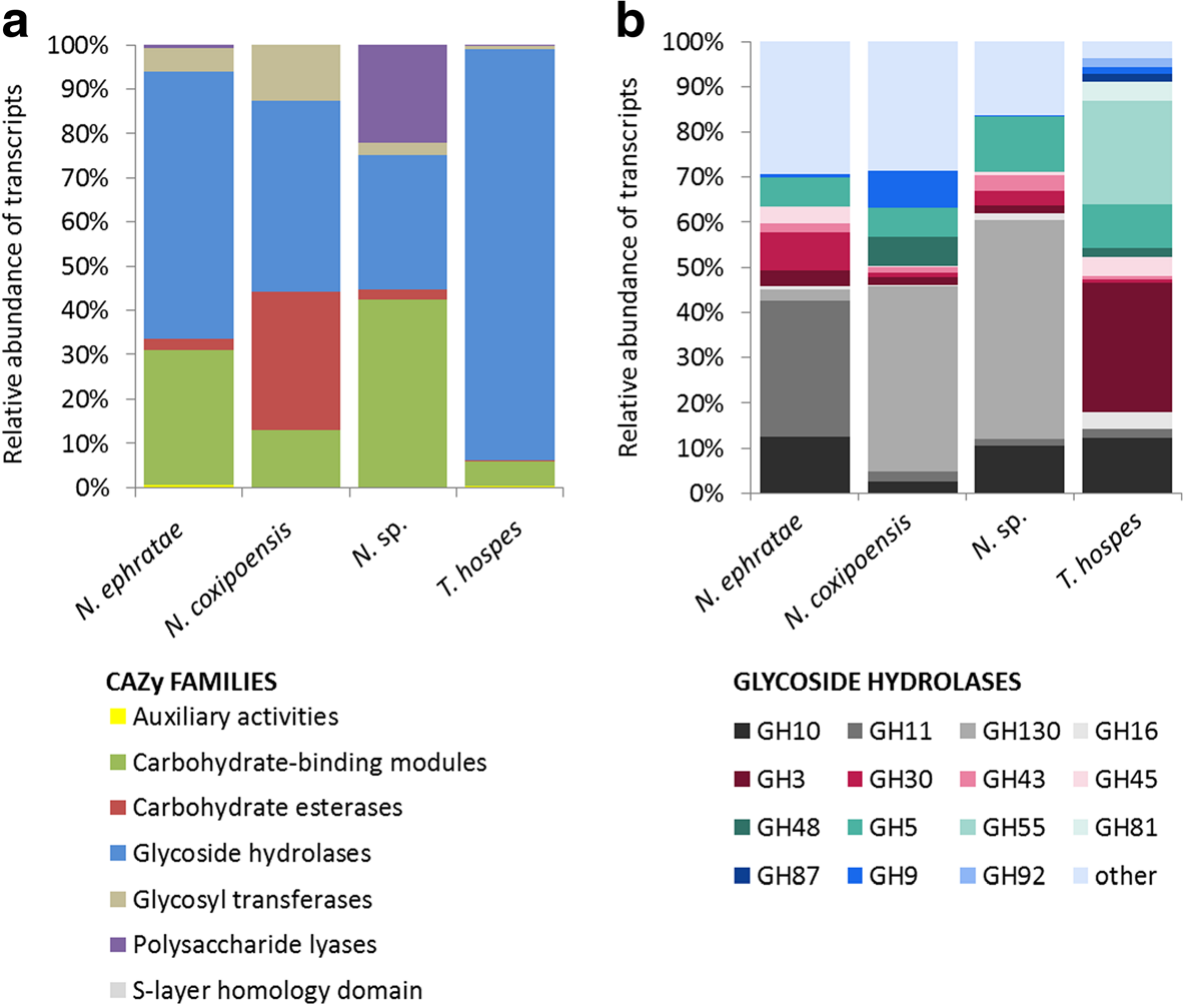
CAZymes in four termite gut microbiomes. **a** Distribution of different CAZymes into classes for the four investigated higher termite gut microbiomes. **b** The most highly expressed glycoside hydrolases for the four investigated higher termite gut microbiomes

In this study, we identified 1643 CAZymes genes transcripts assembled into 1531 contigs that were annotated with significant scores (e-value <1e^−18^ and coverage >0.35) to seven classes of carbohydrate-active enzymes and 116 different families (Fig. 6a and Additional file 4: Table S5). The two most represented CAZymes classes were the glycoside hydrolases (GH; on average 67% ± 27) and the carbohydrate-binding modules (CBM, on average 20% ± 17). In the case of the *N. coxipoensis* metatranscriptome, the class of carbohydrate esterases (CE) was also highly abundant (31%). Among GHs, representatives of the GH130 family (often found to have the phosporylase activity with the affinity for N-glycans) dominated in the *N. coxipoensis* and *N.* sp*.* metatranscriptomes. Family GH3 (putative celluloses and hemicelluloses degrading β-glucosidases and β-xylosidases) as well as GH55 (putative exo- and endo-1,3-glucanases) were mainly represented in the *T. hospes* metatranscriptome. The GH10 family (most probably showing the xylanase activity) and GH5 (broad range of possible activities, e.g. cellulases and hemicellulases) were well represented in all four studied metatranscriptomes (Fig. 6b). Putative xylanases assigned to the family GH11 were highly expressed in the case of *N. ephratae*. Interestingly, CBMs with the predicted affinity towards xylans (families CBM36, CBM13, CBM4, CBM22) dominated the three *Nasutitermes* metatranscriptomes, whereas CBM32 (with previously demonstrated affinity towards galactose, lactose and polygalacturonic acid) and CBM38 (putative affinity towards inulin) dominated the metatranscriptome of *T. hospes*. Additionally, CEs assigned to the CE4 family were highly represented, pointing towards even higher potential for xylan deconstruction in the case of *N. coxipoensis*. Gene transcripts assigned to the family CE15 were the most abundant in the metatranscriptome of *N.* sp. Enzymes classified to this family of CEs (4-O-methyl-glucuronoyl methylesterases) have only recently been discovered in bacteria [55]. Regarding their potential to destroy the covalent linkages connecting lignin and hemicellulose and therefore to detach the lignin from the rest of lignocellulose, they are of major biotechnological interest [56]. Other gene transcripts related to the CAZymes having auxiliary activities (e.g. AA10 belonging to lytic polysaccharide monooxygenases with cellulose depolymerisation potential [57]) and polysaccharide lyases (PLs) from the family PL1 and PL11, were also represented in the four analysed metatranscriptomes. A broad overview of all CAZymes transcripts together with their relative abundances is provided as Additional file 4: Table S5.

The classification of novel CAZymes to already existing families do not necessarily predict their real activity which undoubtedly will have to be determined biochemically for each new enzyme. However, it helps comparing the lignocellulolytic potential of the studied environmental samples, and can help prioritising the interesting enzymes with possibly high expression levels for further verification of their industrial relevance. Further insights into the taxonomic origin (below the domain levels) of the identified symbiotic CAZymes following their phylogenetic assignment were hindered due to very incomplete databases. Their precise taxonomic assignment by homology search was not always possible therefore it will not be discussed in this work. Further omic approaches combining metagenomics and novel microbial genomes reconstruction and metatranscriptomic studies are necessary to unravel the lignocellulose decomposition strategies utilized by the different termite symbiotic bacteria. Given the complexity of the lignocellulosic substrates, and the fact that over millions of years different termite species have adapted and optimized their digestive tracks to a variety of lignocellulosic complexes, the exploration of new biological diversity/functions will allow us to better understand, and in continuation to effectively mimic these efficient lignocellulolytic systems.

## Conclusions

Although the biorefinery of biomass to biofuels is a man-made concept, and is not widespread in nature, natural organisms/systems can effectively mediate the different steps in the course of the process. Some organisms, e.g. higher termites surviving on lignocellulose biomass, developed different adaptations including their specialized gut systems harbouring diverse microbiota. Therefore, the examination of the termite gut symbiotic microbes should reveal interesting enzymes capable of hydrolysing a broad range of chemical bonds, which is of primary interest for the industry. While recent metatranscriptomic reports showed the high representation and overexpression of cellulose and hemicelluloses degrading gene transcripts in the termite hindgut, several factors associated with the material sampling and library preparation, make the metatranscriptomic studies of higher termite gut challenging.

Here, we have covered different aspects, including the termite gut sampling strategy (WG versus LF) and the prokaryotic mRNA enrichment pipelines (ME versus RZ), with the view of optimising the metatranscriptomics of the higher termite symbiotic bacteria. As a result, we have shown that the sampling strategy does not significantly influence the resulting metatranscriptomic profiles of termite gut microbes. However, the combination of the LF sampling with the RZ library preparation pipeline results in a significantly increased sequencing depth of bacterial mRNA transcripts, at the same time depleting up to 98.4% of residual rRNAs and 97.9% poly A-mRNAs can be preserved and sequenced separately if of interest). As a final goal, using our optimised approach, we compared the diversity of CAZymes in the metatranscriptomes of the gut symbiotic communities of the four termite species targeted in this study (*N. coxipoensis, N. ephratae, N.* sp. and *T. hospes*). The COG category related to carbohydrate transport and metabolism was the second most highly represented, indicating relatively high expression levels of different CAZymes in relation to other mRNA transcripts present in the metatranscriptome of the higher termite gut symbionts.

The methodology proposed here offers a highly efficient and accurate framework to study the metatranscriptomes of the higher termite symbiotic communities, with a focus on novel CAZymes. Since metatranscriptomics directly provides the data describing the overall transcriptomic levels, it will also provide new knowledge to the scientific community for future characterization of other novel metabolic pathways/activities of the termite system. Moreover, the methodology can be useful to explore other lignocellulose-degrading systems as well.

## Additional files

Additional file 1: Table S1. Overview of termite nests used in the study. (XLSX 10 kb)

Additional file 2: Bioanalyser results for the total RNA extractions after the TURBO DNAse treatment for: **Figure S1.** (A) N_eph_LF, (B) N_eph_WG, **Figure S2.** (A) N_cox_LF, (B) N_cox_WG, **Figure S3:** (A) N_sp_LF, (B) N_sp_WG, **Figure S4.** (A) T_hos_LF, (B) T_hos_WG. (DOCX 3703 kb)

Additional file 3: Figure S5. Overview of the bioinformatic pipeline applied in this study to analyse metatranscriptomic libraries of termite gut symbiotic bacteria. (DOCX 369 kb)

Additional file 4: Table S2. Overview of the sequencing statistics, **Table S3:** Levels of rRNA reads detected by SortMeRNA software, **Table S4:** Levels of transcripts annotated to protein coding genes, **Table S5:** Broad overview of all transcripts with significant annotation to CAZymes together with relative abundances. (XLS 273 kb)

Additional file 5: Figure S6. The observed richness estimator rarefaction curves based on high-throughput amplicon sequencing of 16S rRNA gene for eight tested samples of termite gut bacteria. **Table S6.** Observed richness and diversity metrics for the eight tested samples of termite gut bacteria. (DOCX 326 kb)

Additional file 6: Figure S7. The average representation of gene transcripts annotated to COG categories for the four tested termite microbiomes. (DOCX 222 kb)

## Abbreviations

AA: Auxiliary activities
CAZymes: Carbohydrate-active enzymes
CBM: Carbohydrate-binding modules
CE: Carbohydrate esterases
COG: Clusters of orthologous groups of proteins
CO-II: Cytochrome oxidase subunit 2
Contig: Contiguous sequence
GH: Glycoside hydrolases
GT: Glycosyl transferases
LF: Luminal fluid
ME: Prokaryotic mRNA enrichment pipeline “Pipeline ME”
N_eph: *Nasutitermes ephratae*
N_sp: *Nasutitermes* sp.
ND: Non-depleted
NMDS: Non-metric multidimensional scaling
OTU: Operational taxonomic unit
PBS: Phosphate Buffered Saline
PCR: Polymerase chain reaction
PL: Polysaccharide lyases
qPCR: Quantitative polymerase chain reaction
RT: Room temperature
RZ: Prokaryotic mRNA enrichment pipeline “Pipeline RZ”
SLH: S-layer homology domain
T_hos: *Termes hospes*
WG: Whole gut
