# Supplementary material for: Optimization of a metatranscriptomic approach to study the lignocellulolytic potential of the higher termite gut microbiome
Source: BMC Genomics. 2017 Sep 1;18:681. doi: 10.1186/s12864-017-4076-9 (PMC5580439; doi:10.1186/s12864-017-4076-9)
Supplement: Supplementary file 2 — Bioanalyser results for the total RNA extractions after the TURBO DNAse treatment for: Figure S1. (A) N_eph_LF, (B) N_eph_WG, Figure S2. (A) N_cox_LF, (B) N_cox_WG, Figure S3: (A) N_sp_LF, (B) N_sp_WG, Figure S4. (A) T_hos_LF, (B) T_hos_WG. (DOCX 3703 kb) [file 12864_2017_4076_MOESM2_ESM.docx]

**Additional file 2**

**
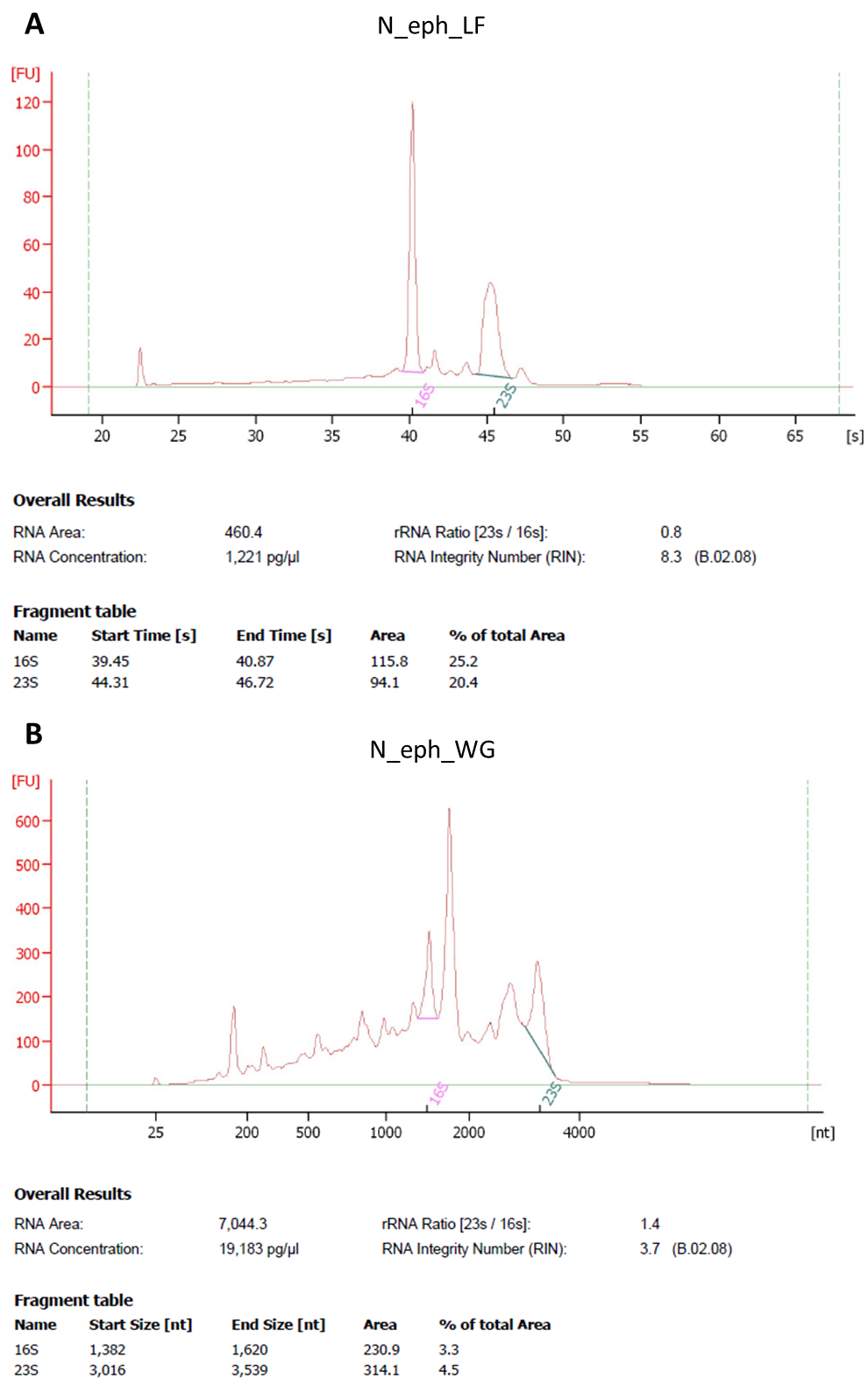
**

**Figure S1**

Bioanalyser results for the total RNA extractions after the TURBO DNAse treatment for **(A)** N_eph_LF and **(B)** N_eph_WG.


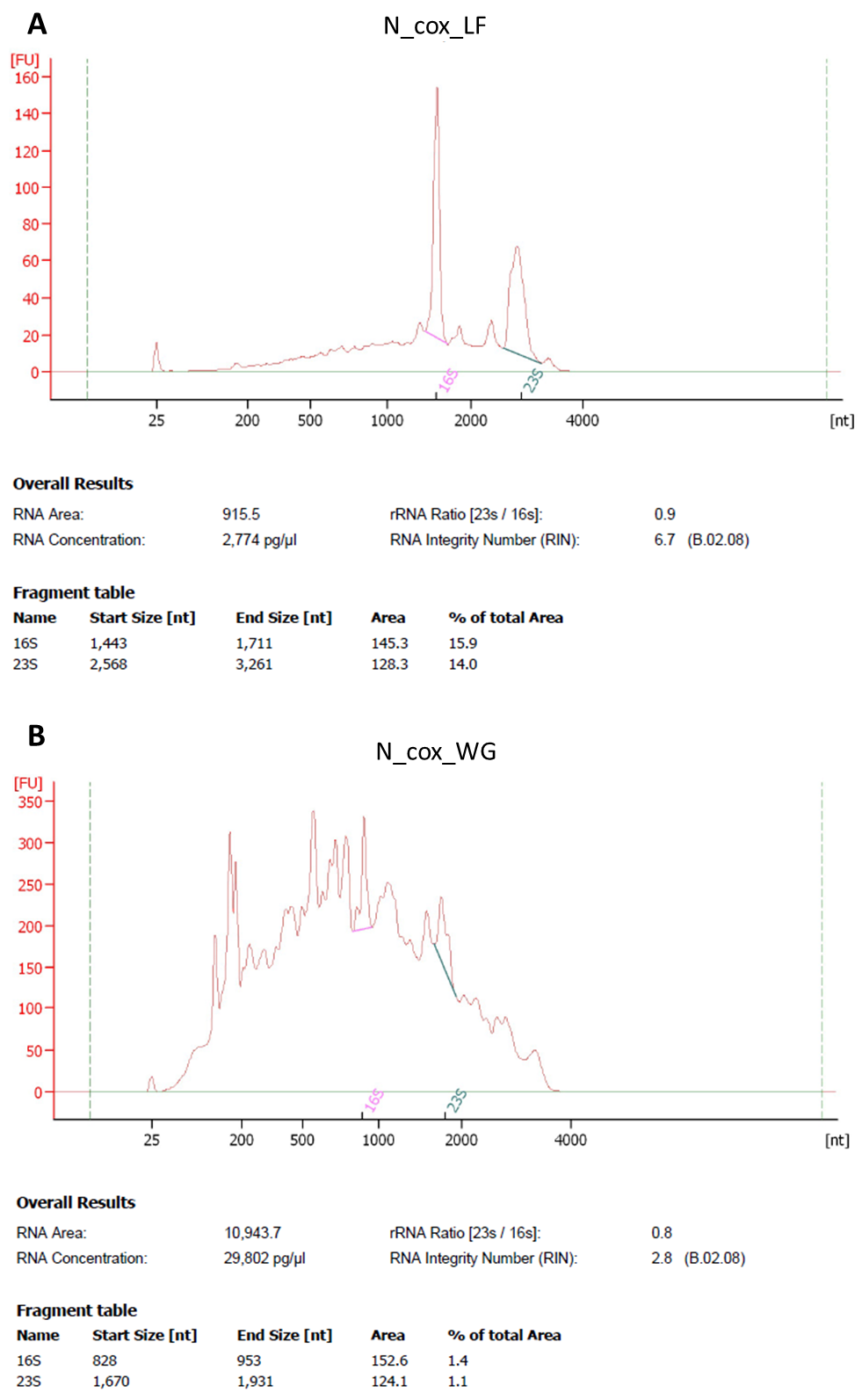


**Figure S2**

Bioanalyser results for the total RNA extractions after the TURBO DNAse treatment for **(A)** N_cox_LF and **(B)** N_cox_WG.


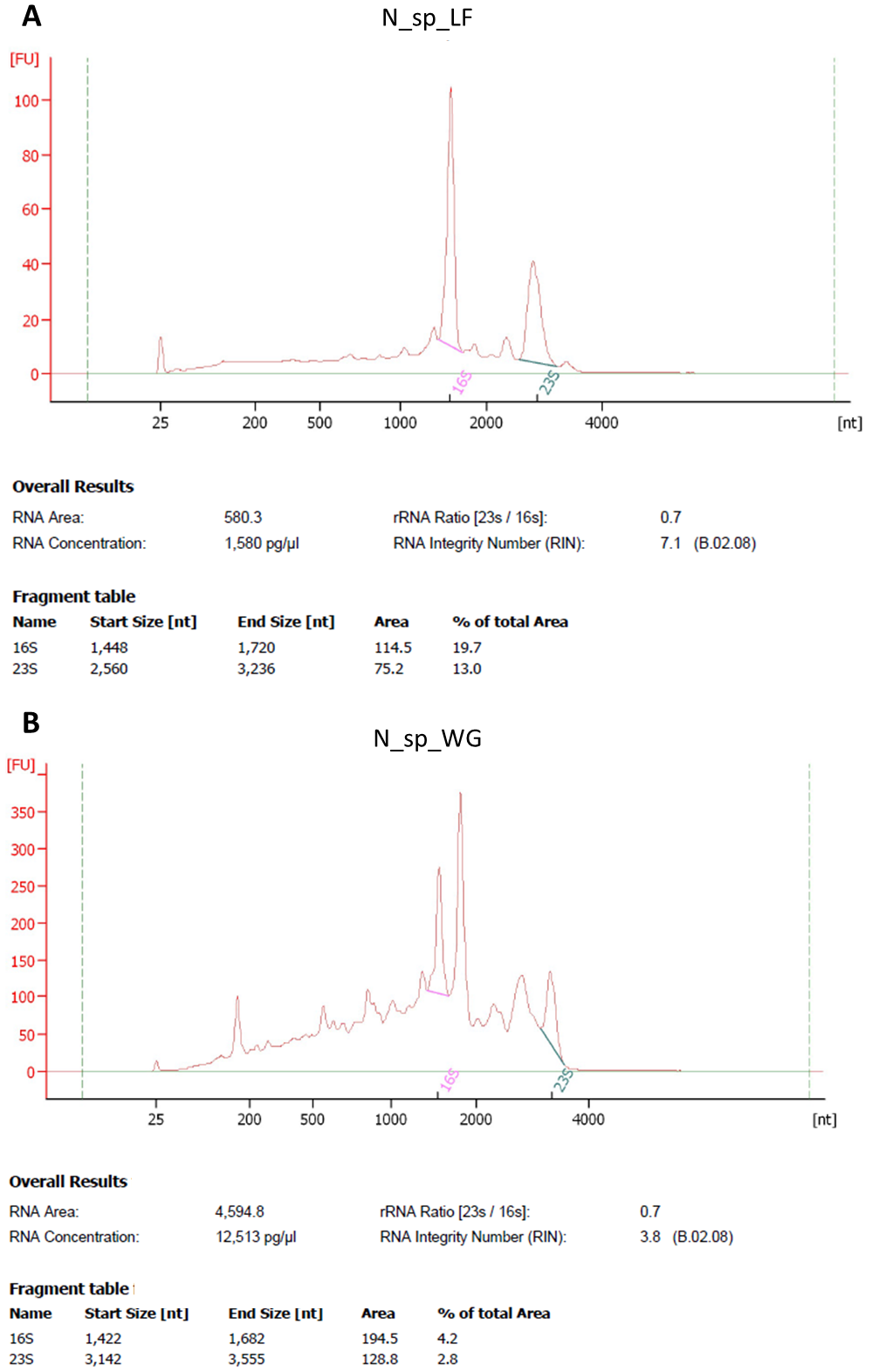


**Figure S3**

Bioanalyser results for the total RNA extractions after the TURBO DNAse treatment for **(A)** N_sp_LF and **(B)** N_sp_WG.


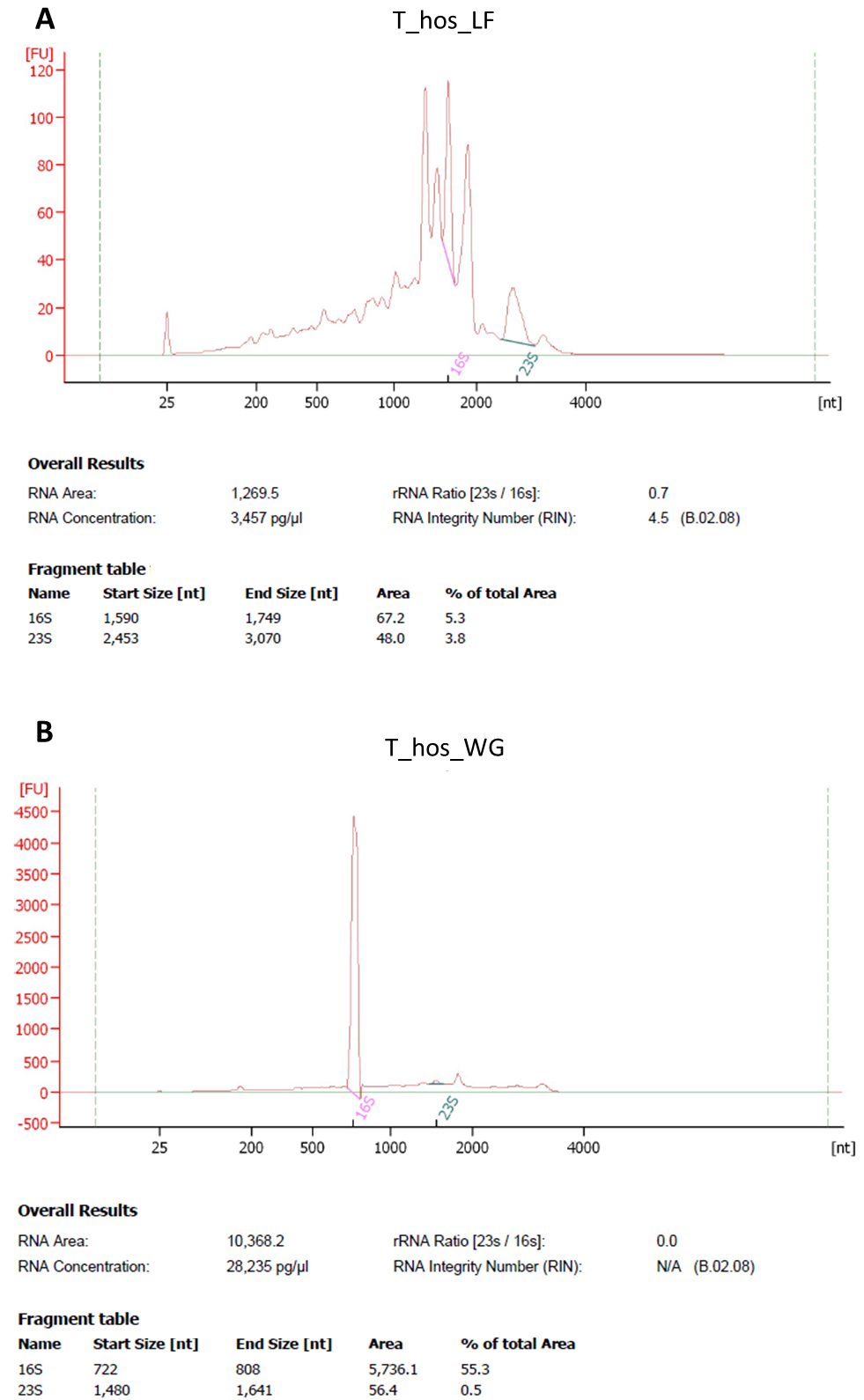


**Figure S4**

Bioanalyser results for the total RNA extractions after the TURBO DNAse treatment for **(A)** T_hos_LF and (B) T_hos_WG.
