# Supplementary material for: Optimization of a metatranscriptomic approach to study the lignocellulolytic potential of the higher termite gut microbiome
Source: BMC Genomics. 2017 Sep 1;18:681. doi: 10.1186/s12864-017-4076-9 (PMC5580439; doi:10.1186/s12864-017-4076-9)
Supplement: Supplementary file 3 — Overview of the bioinformatic pipeline applied in this study to analyse metatranscriptomic libraries of termite gut symbiotic bacteria. (DOCX 369 kb) [file 12864_2017_4076_MOESM3_ESM.docx]

**Additional file 3**


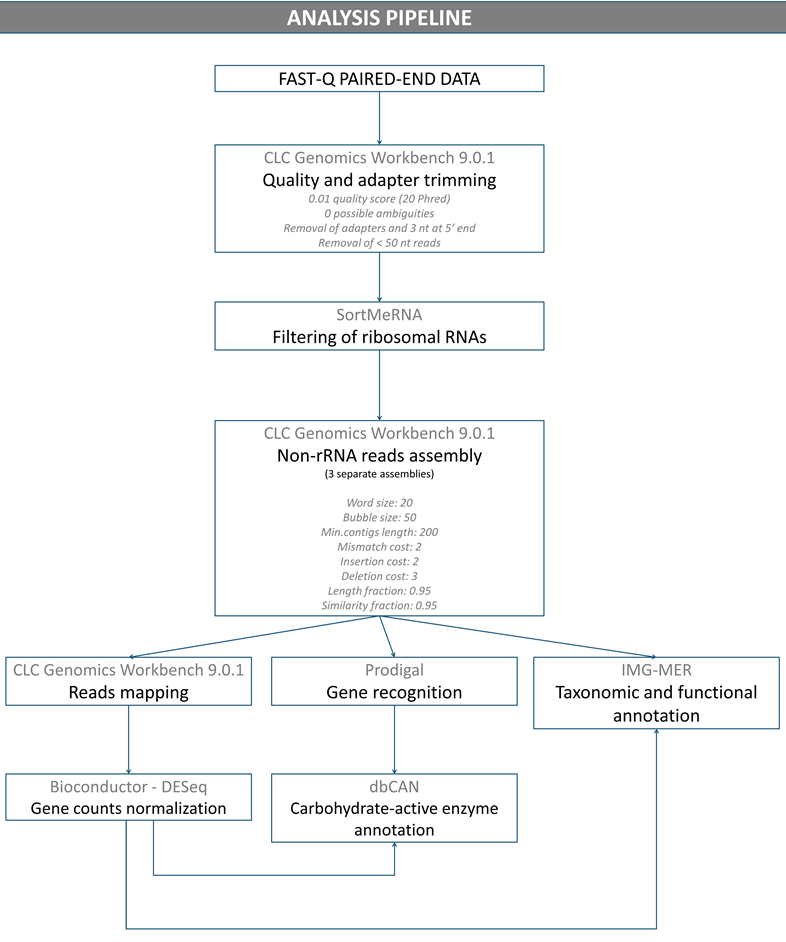


**Figure S5**

Overview of the bioinformatic pipeline applied in this study to analyse metatranscriptomic libraries of termite gut symbiotic bacteria.
