# Supplementary material for: Optimization of a metatranscriptomic approach to study the lignocellulolytic potential of the higher termite gut microbiome
Source: BMC Genomics. 2017 Sep 1;18:681. doi: 10.1186/s12864-017-4076-9 (PMC5580439; doi:10.1186/s12864-017-4076-9)
Supplement: Supplementary file 5 — The observed richness estimator rarefaction curves based on high-throughput amplicon sequencing of 16S rRNA gene for eight tested samples of termite gut bacteria. Table S6. Observed richness and diversity metrics for the eight tested samples of termite gut bacteria. (DOCX 326 kb) [file 12864_2017_4076_MOESM5_ESM.docx]

**Additional file 5**


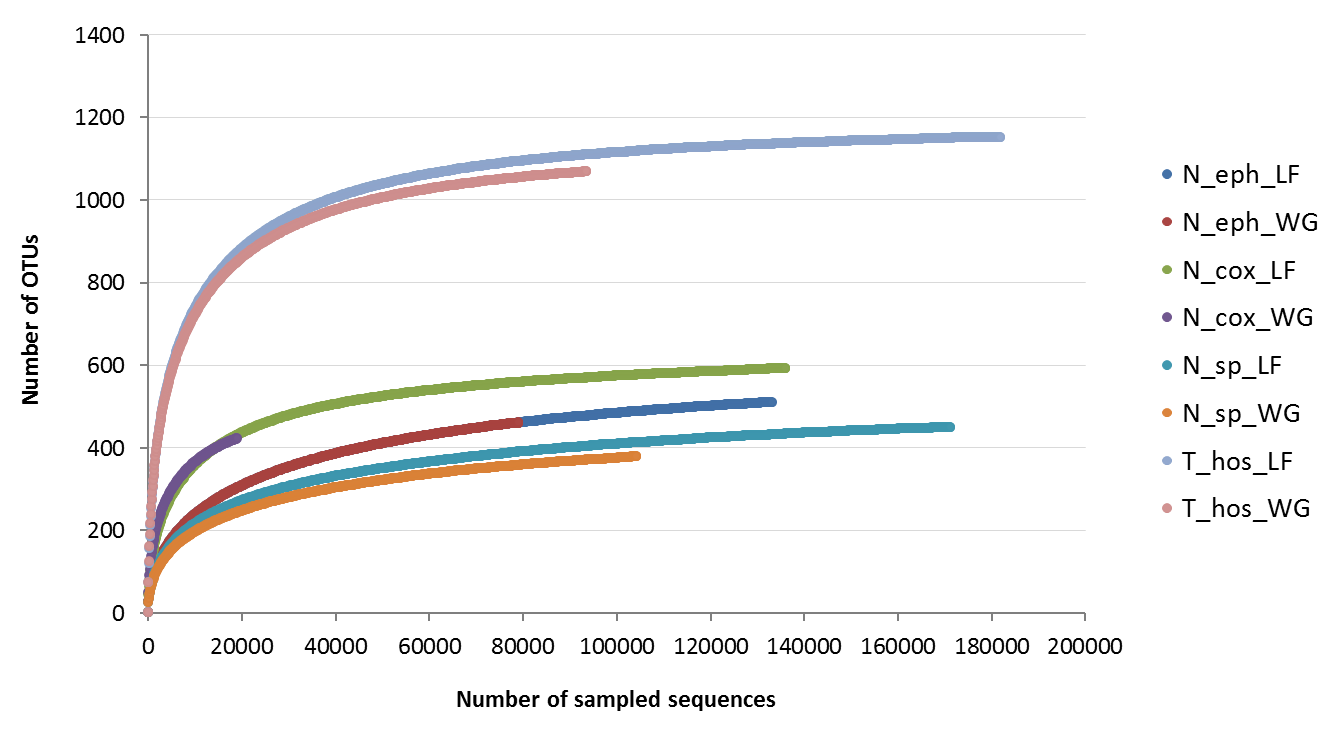


**Figure S6**

The observed richness estimator rarefaction curves based on high-throughput amplicon sequencing of 16S rRNA gene for eight tested samples of termite gut bacteria.

**Table S6** Observed richness and diversity metrics for the eight tested samples of termite gut bacteria.

| Sample | Sobs^1^ | Invsimpson^2^ | invsimpson_lci^3^ | invsimpson_hci^3^ |
| --- | --- | --- | --- | --- |
| N_cox_LF | 592 | 28.0 | 27.7 | 28.3 |
| N_cox_WG | 422 | 28.0 | 27.1 | 29.0 |
| N_eph_LF | 510 | 14.4 | 14.3 | 14.5 |
| N_eph_WG | 371 | 13.6 | 13.5 | 13.8 |
| N_sp_LF | 450 | 9.2 | 9.2 | 9.3 |
| N_sp_WG | 378 | 9.3 | 9.2 | 9.4 |
| T_hos_LF | 1152 | 93.8 | 92.6 | 95.1 |
| T_hos_WG | 1068 | 113.0 | 111.0 | 115.0 |

^1^Observed richness (number of observed OTUs) calculated for 8 tested samples (Sobs calculator).

^2^Invsimpson – alpha-diversity estimation calculated for eight tested samples (invsimpson calculator).

^3^lci and hci are rarefied 95% lower and higher confidence intervals provided by *mothur* software
