# Supplementary material for: Optimization of a metatranscriptomic approach to study the lignocellulolytic potential of the higher termite gut microbiome
Source: BMC Genomics. 2017 Sep 1;18:681. doi: 10.1186/s12864-017-4076-9 (PMC5580439; doi:10.1186/s12864-017-4076-9)
Supplement: Supplementary file 6 — The average representation of gene transcripts annotated to COG categories for the four tested termite microbiomes. (DOCX 222 kb) [file 12864_2017_4076_MOESM6_ESM.docx]

**Additional file 6**


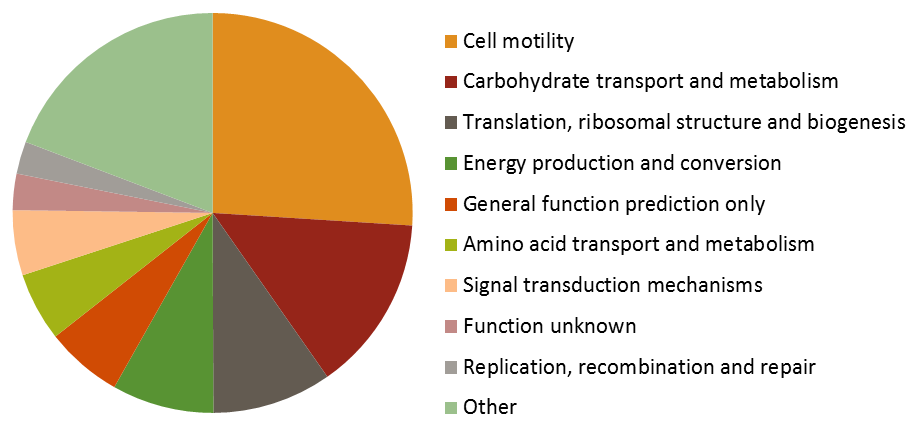


**Figure S7**

The average representation of gene transcripts annotated to COG categories for the four tested termite microbiomes.
